# Visceral adipose tissue and carotid intima-media thickness in HIV-infected patients undergoing cART: a prospective cohort study

**DOI:** 10.1186/s12879-017-2884-9

**Published:** 2018-01-11

**Authors:** Maria Teresa Beires, André Silva-Pinto, Ana Cristina Santos, António José Madureira, Jorge Pereira, Davide Carvalho, António Sarmento, Paula Freitas

**Affiliations:** 1 Faculty of Medicine, University of Porto. Alameda Prof. Hernâni Monteiro, 4200-319 Porto, Portugal; 2 Infectious Diseases Department, Centro Hospitalar São João, Porto, Portugal; 3 Renal, Urological and Infectious Diseases Department, Faculty of Medicine of University of Porto, Porto, Portugal; 4 EPIUnit - Instituto de Saúde Pública, Universidade do Porto, Porto, Portugal; 5 Departamento de Ciências da Saúde Pública e Forenses e Educação Médica, Faculdade de Medicina, Universidade do Porto, Porto, Portugal; 6 Radiology Department, Hospital de São João and University of Porto Medical School, Porto, Portugal; 7 Nuclear Medicine Department, Hospital de São João, Porto, Portugal; 8 Endocrinology Department, Hospital de São João and University of Porto Medical School, Porto, Portugal

**Keywords:** Lipodystrophy, HIV, Carotid intima media thickness, Fat mass ratio

## Abstract

**Background:**

Combined antiretroviral therapy (cART) in HIV-infected patients has been associated with lipodystrophy, metabolic abnormalities, and an increased risk of cardiovascular disease. Ultrasound measures of carotid artery intima-media thickness (cIMT) have been used as a valid measure of subclinical atherosclerosis and as a tool to predict the risk of cardiovascular events. Our aim was to evaluate the progression of cIMT in HIV-infected patients subjected to cART, with and without lipodystrophy, over a one-year period.

**Methods:**

We performed a one-year prospective cohort study to compare changes in cIMT, metabolic and inflammation markers in HIV-infected patients undergoing cART. Body composition was assessed by dual-energy X-ray absorptiometry (DXA) and abdominal computed tomography (CT). Levels of blood pressure, lipids and inflammatory markers were evaluated, as well as ultrasound measures of cIMT. Lipodystrophy defined by Fat Mass Ratio (L-FMR) is measured as the ratio of the percentage of trunk fat mass to the percentage of lower limb fat mass by DXA. Categorical variables were compared, using the chi-square or Fisher’s exact test. Wilcoxon ranks tests and the McNemar chi-square tests were used to compare results of selected variables**,** from the first to the second year of evaluation. Means of cIMT, adjusted for age, glucose, triglycerides levels, systolic blood pressure (SBP), and waist to hip ratio were calculated, using generalised linear models for repeated measures.

**Results:**

L-FMR was present in 44.3% of patients, and the mean of cIMT increased significantly in this group [0.82 (0.26) vs 0.92 (0.33); *p* = 0.037], as well as in patients without lipodystrophy [0.73 (0.20) vs 0.84 (0.30); *p* = 0.012]. In the overall sample, the progression of cIMT was statistically significant after the adjustment for age, glucose, triglycerides, and SBP, but the significance of the progression ceased after the adjustment for waist/hip ratio [0.770 (0.737–0.803) vs 0.874 (0.815–0.933); *p* = 0.514].

**Conclusions:**

Carotid IMT progressed significantly in both groups of this HIV-infected cohort, however no association between the progression of cIMT and the presence of lipodystrophy defined by FMR was found. Visceral adipose tissue had an impact on the increment of cIMT, both in patients with, and without lipodystrophy defined by FMR.

## Background

The use of combined antiretroviral therapy (cART) in HIV-infected patients has significantly increased the life expectancy of these subjects [1]. However, as a consequence, cardiovascular disease (CVD) has emerged as being an important late concern in HIV-infected patients subjected to this treatment [2]. It has been proven that HIV-infected patients treated with cART have an increased risk of developing cardiovascular disease, and thus it became essential to understand the underlying mechanism associated with this outcome. HIV-lipodystrophy is a well-established side effect of cART, especially when associated with some specific regimens [3]. Patients with lipodystrophy may present a higher risk of premature atherosclerosis, as abnormalities in body fat distribution are associated with various metabolic risk factors that lead to cardiovascular disease [4].

The relative contributions of conventional cardiovascular risk factors, metabolic side effects of antiretroviral drugs, and HIV infection itself on cardiovascular risk are difficult to identify, as these factors frequently occur simultaneously [5]. In this context, intima-media thickness of the carotid arteries (cIMT) has become a surrogate marker for atherosclerosis and it has shown to be an independent risk factor for the unfolding cardiovascular disease, on the grounds that it can be accurately and safely measured by ultrasound [6].

In a previously published study, we showed that HIV-infected patients under cART with lipodystrophy defined by FMR had a significantly higher cIMT than those without lipodystrophy, which was also associated with classical cardiovascular risk factors, such as visceral adipose tissue and age [6]. The purpose of this study was to evaluate the progression of atherosclerosis assessed by cIMT in the total of the HIV-infected patients undergoing cART, and in those with or without lipodystrophy defined by Fat Mass Ratio (L-FMR), and to evaluate the evolution of established cardiovascular risk factors during one year of follow-up.

## Methods

### Subjects

We evaluated 115 HIV-infected patients undergoing cART in this longitudinal study. Only non-institutionalised caucasian adults who were subjected to cART were evaluated, and all patients were referred from the Infectious Diseases Outpatient Clinic. We excluded institutionalised patients with the objective of ruling out acute intercurrences that could have an impact on the metabolic and inflammatory parameters. Participants were evaluated at baseline, and after one year for each parameter (clinical assessment, evaluation of body composition, laboratory analysis, and carotid IMT measurements). All patients were referred to a nutrition appointment, were motivated to comply with a structured diet plan, walk or exercise regularly and were also advised to stop smoking. Overweight patients, or those suffering from obesity were motivated to lose weight. Prediabetes, diabetes, hypertension, and dyslipidemia were treated according to the standard of care for the HIV-population. This study was approved by the Ethics Committee for Health of the Hospital São João in Porto and each patient provided their written, informed consent.

### Clinical assessment

For each patient, the following information was collected at both baseline and after the follow-up period (12 months), using a standard protocol that covered: age, gender, known duration of HIV infection, and duration of cART exposure, HIV risk factor, characterisation of the infection, smoking history, family history of cardiovascular diseases, and use of anti-hypertensive, anti-diabetic, or lipid lowering drugs.

We used the “Centers for Disease Control and Prevention” (CDC) HIV staging classification [7], whereby measurements of weight, height, neck, waist, hip, thigh and arm circumference were performed as previously described [8] and were carried out by the same observer, in a standard fashion [9].

Measures of blood pressure were taken with the patient in a supine position, employing a standardised technique, as previously described [10].

### Evaluation of body composition

We evaluated body composition using whole-body, dual-energy X-ray absorptiometry (DXA – Lunar Expert XL, 1999). DXA measurements were carried out whilst in a supine position, and standard positioning of the arms and feet was established according to the manufacturer’s instructions. Regional fat mass values were grouped and analyzed for the following anatomical regions: arms, legs, trunk, and total body. The fat mass ratio (FMR) was measured as the ratio between the percentage of trunk fat mass and the percentage of lower limb fat mass (FMR = % of the trunk fat mass/% of the lower limb fat mass) [11]. We used a cut-off value for lipodystrophy defined by FMR for men of 1.961, and 1.329 for women [12].

The quantification of total, visceral, and subcutaneous fat was performed with a 64-slice, abdominal computed tomography (CT) scanner (Siemens Sensation 64 Cardiac), with the same technique as previously described [13, 14]. All values were expressed in cm^2^, rounded to the nearest centesimal.

### Laboratory analysis

#### Biological and inflammatory parameters

A venous blood sample was taken after a 12 h overnight fast. All the samples were analyzed at the central laboratory of our hospital. The measurements of total cholesterol (TC), low density lipoprotein (LDL) cholesterol, high-density lipoprotein (HDL) cholesterol, triglycerides, apolipoprotein A1 (Apo A1), apolipoprotein B (Apo B), lipoprotein (a) [Lp (a)], fibrinogen, high sensitivity C-reactive protein (hsCRP), homocysteine, uric acid, lactates, NT-proBNP, glucose, insulin, and A1c serum levels were determined using commercial kits. Non-HDL-C was defined as TC-HDL. Microalbuminuria was determined in a 24-h urine sample. Patients without a previous diagnosis of diabetes were submitted to an oral glucose tolerance test (OGTT). This test was performed as instructed by the World Health Organisation [15].

The CD4+ cell count (×10^6^ cell/L) was determined by flow cytometry and plasma HIV-1 RNA loads were measured by a quantitative reverse transcriptase polymerase chain reaction (Roche Diagnostic Systems, Inc., Branchburg, NJ, USA), which had a lower limit of detection of 50 copies/mL.

Insulin resistance was defined by the homeostasis model assessment of insulin resistance (HOMA), using the following formula:

HOMA – IR index = (insulin 0 x glucose 0)/22.5 [16].

We defined Metabolic Syndrome (MS) according to the modified NCEP-ATP III criteria, requiring the presence of three of the following risk factors: (1) elevated waist circumference (>102 cm); (2) elevated triglycerides (≥150 ml/dL or specific treatment); (3) reduced HDL-C levels (<40 mg/dL); (4) elevated blood pressure (>130/ 85 mmHg or treatment with anti-hypertensive drugs), and; (5) elevated fasting glucose (≥100 mg/dL or use of glucose-lowering drugs). [17]

### Carotid IMT measurements

We performed high-resolution B-mode and Doppler ultrasounds measures of the carotid arteries using a Philips iU22 machine (Philips Medical, The Netherlands), equipped with a 17–5 MHz high-frequency linear-array transducer. Patients were examined in the supine position, with the head in a neutral position, or slightly turned away from the side that was being scanned. The left and right common carotid arteries (CCA) were examined in multiple directions. The carotid bifurcation was included in the image plane to serve as a landmark and provide accurate serial measurements. The IMT was measured along a segment of the artery free of atherosclerotic plaque with clearly defined lumen-intima and media-adventitia interfaces if possible 3 cm below the carotid bulb. The far wall was preferred for the measurement and only when the image quality of the far wall was not satisfactory was the near wall used. The measurements were done by manual tracking and were repeated three times. The mean IMT of these values was calculated. All studies were performed by the same radiologist, who had 12 years of experience in vascular ultrasound, and were carried out using the same machine.

The presence of subclinical carotid atherosclerosis was defined as IMT > 0.80 mm, the presence of plaque, or both [18–20].

### Statistical analysis

Data were described as the mean and standard deviation (SD) for quantitative variables and were compared using Student-t or Mann-Whitney tests, as appropriate. Categorical variables were described as counts and proportions, and compared using the chi-square or Fisher’s exact test. To study the association between cIMT and clinical and metabolic characteristics, Spearman correlation coefficients were estimated. In addition, Wilcoxon ranks tests and the McNemar chi-square tests were used to compare results of selected variables from the baseline to the first follow-up evaluation. Means of cIMT were calculated, using generalised linear models for repeated measures, adjusted for age, glucose, triglycerides levels, systolic blood pressure, and waist to hip ratio.

Statistical analysis was performed using SPSS version 24.0 software (SPSS Inc., Chicago, Illinois, USA). All probabilities were two-tailed, and *p* values of <0.05 were regarded as significant.

## Results

### Baseline

#### Patient characteristics, body composition by DXA and metabolic parameters.

In 115 (91 male and 24 female) HIV-infected patients undergoing cART, 51 (44.3%) presented lipodystrophy defined by FMR. The characteristics of the study sample according to the presence of lipodystrophy defined by FMR are illustrated in Table 1. Patients with lipodystrophy had been infected with HIV for a longer period of time (*p* = 0.036) and were subjected to a greater length of cART (*p* = 0.001). No differences in weight, height, BMI, waist, neck, and thigh and arm circumferences among patients with or without lipodystrophy were found.

**Table 1:** Basal sample characteristics according to the presence of lipodystrophy defined by FMR

	Without Lipodystrophy defined by FMR	With Lipodystrophy defined by FMR	*P*
n (%)	64	51	–
Gender [n(%)]			
Male	49 (76.6%)	42(82.4%)	0.448
Female	15(23.4%)	9(17.6%)	
Age [years, mean (sd)]	45 (12.2)	49(9.9)	0.052
Duration of HIV infection [years, mean (sd)]	7.2 (3.8)	8.5(3.7)	0.036
cART [years, mean (sd)]	5.9 (3.6)	7.9 (3.5)	0.001
Weight [Kg, mean (sd)]	69.5 (13.5)	67.8 (9.6)	0.410
Height [m, mean (sd)]	1.66 (0.09)	1.64 (0.08)	0.188
BMI [(Kg/m^2^), mean (sd)]	25.2 (4.7)	25.2 (3.8)	0.976
Waist circumference [cm, mean (sd)]	91.3 (12.7)	91.6 (9.9)	0.877
Hip circumference [cm, mean (sd)]	95.7(8.5)	91.9(6.4)	0.006
Thigh circumference [cm, mean (sd)]	48,7 (5.1)	47.1(5.2)	0.113
Arm circumference [cm, mean (sd)]	26.9 (3.0)	27.0 (2.7)	0.939
Neck circumference [cm, mean (sd)]	37.8 (3.6)	37.6 (3.3)	0.829
Waist/ hip circumference ratio [mean (sd)]	0.95 (0.09)	0.99 (0.07)	0.003
CD4 cell count [cells/mm3, mean (sd)]	522.9 (288.7)	584.6 (331.9)	0.289
HIV RNA (<50) [n (%)]	52 (81.3%)	47 (92.92%)	0.093
HIV risk factor [n (%)]			
Injecting drug user	19 (29.7%)	11 (21.6%)	0.073
Homosexual contact	39 (60.9%)	27 (52.9%)	
Heterosexual contact	4 (6.3%)	11 (21.6%)	
Others	1(1.6%)	2 (3.9%)	
CDC [n (%)]			
A	32 (50.0%)	31(60.8%)	0.248
B	–	–	
C	32 (50.0)	20 (39.2)	
ART [n (%)]			
PI	32 (50.0%)	30 (58.8%)	0.346
NNRTI	31 (48.4%)	23 (45.1%)	0.721
NRTI	60 (93.8%)	50 (98.0%)	0.380
Smoking history [n (%)]			
Never	19 (30.2%)	20 (39.2)	0.556
Current	33 (52.4%)	22 (43.1%)	
Former	11 (17.5%)	9 (17.6%)	
Familial history of CVD [n (%)]	23 (35.9%)	21 (41.2%)	0.566
Taking medications [n (%)]			
Statins	14 (22.2%)	15 (29.4%)	0.381
Fibrates	29 (46.0%)	27 (52.9%)	0.463
Oral anti-diabetics	9 (14.3%)	12 (23.5%)	0.306
Insulin	4 (6.3%)	4 (7.8%)	0.999
Anti-hypertensive drugs	13 (20.3%)	12 (24.0%)	0.637
Fat mass [%, mean (sd)] DXA			
Total	22.6 (13.1)	17.8 (8.0)	0.017
Trunk	24.1 (13.5)	22.7 (8.6)	0.508
Leg	22.3 (15.4)	9.7 (6.7)	<0.001
Arm	23.4 (16.3)	18.7 (13.3)	0.095
Fat mass [Kg, mean (sd)] DXA			
Total	16.4 (11.0)	12.1 (6.6)	0.012
Trunk	9.1 (6.2)	8.4 (4.3)	0.470
Leg	4.7 (3.4)	1.8 (1.3)	<0.001
Arm	1.9 (1.7)	13.8 (1.2)	0.063
Body Fat Mass by Quantitative CT			
Total fat [cm^2^, mean (sd)]	272.6 (188.1)	259.6 (112.9)	0.651
VAT [cm^2^, mean (sd)]	116.8 (95.4)	154.8 (59.7)	0.011
SAT [cm^2^, mean (sd)]	155.8 (123.2)	104.8 (85.2)	0.011
VAT/SAT ratio [cm^2^, mean (sd)]	1.3 (1.8)	2.4 (1.7)	0.002
Systolic blood pressure [mmHg, mean (sd)]	122.0 (19.4)	124.0 (16.0)	0.558
Diastolic blood pressure [mmHg, mean (sd)]	78.5 (12.1)	79.2 (11.1)	0.750
Leukocytes [10^9^/L, mean (sd)]	5.7 (1.7)	6.5 (1.9)	0.034
Glucose 0 min [mg/dL, mean (sd)]	106.4 (47.1)	119.1 (52.1)	0.175
Glucose 2 h [mg/dL, mean (sd)]	122.3 (46.3)	138.5 (47.7)	0.126
Insulin 0 min [μU/mL, mean (sd)]	10.5 (12.8)	10.9 (6.5)	0.863
Insulin 2 h [μU/mL, mean (sd)]	53.1 (49.2)	105.9 (186.8)	0.072
HOMA [mean (sd)]	2.9 (3.6)	3.2 (2.6)	0.601
A1c [% mean (sd)]	5.6 (1.2)	6.0 (1.1)	0.123
Total cholesterol [mg/dL, mean (sd)]	213.6 (56.0)	226.9 (57.6)	0.216
LDL-cholesterol [mg/dL, mean (sd)]	113.5 (49.2)	123.7 (49.2)	0.270
HDL-cholesterol [mg/dL, mean (sd)]	43.9 (12.1)	43.6 (11.3)	0.911
Non-HDL cholesterol [mg/dL, mean (sd)]	169.8 (52.0)	183.2 (52.2)	0.170
Triglycerides[mg/dL, mean (sd)]	284.4 (191.7)	298.9 (186.9)	0.683
Apo A1 [mg/dL, mean (sd)]	110.4 (24.0)	111.6 (16.6)	0.763
Apo B [mg/dL, mean (sd)]	104.7 (24.4)	104.8 (26.7)	0.975
Ratio apo B/apo A1 [mean (sd)]	0.98 (0.3)	0.95 (0.3)	0.604
Lp (a) [mg/dL, mean (sd)]	28.9 (31.0)	27.5 (33.5)	0.821
Homocysteine [μmol/L, mean (sd)]	9.4 (4.1)	9.7 (3.5)	0.701
CRP [mg/L, mean (sd)]	6.6 (16.6)	4.3 (4.7)	0.496
hsCRP [mg/dL, mean (sd)]	0.83 (1.5)	0.34 (0.31)	0.496
Lactates [mmol/L, mean (sd)]	1.1 (0.5)	1.3 (0.6)	0.056
NT-ProBNP [pg/mL, mean (sd)]	41.2 (74.8)	31.6 (31.9)	0.450
Fibrinogen [mg/dL, mean (sd)]	350.8 (103.8)	342.9 (105.9)	0.696
Microalbumin [mg/L, mean (sd)]	76.7 (196.6)	40.6 (91.3)	0.441
Uric acid [mg/L, mean (sd)]	46.0 (14.6)	49.8 (12.8)	0.140
Carotid IMT [mm, mean (sd)]	0.73 (0.196)	0.82 (0.257)	0.050
Metabolic Syndrome (%)	31 (49.2%)	27 (54.0%)	0.705

(*L* Lipodystrophy FMR defined, *CDC* Centers for Disease Control and Prevention criteria for staging of HIV infection, *cART* Combined antiretroviral therapy, *BMI* Body mass index, *FMR* Fat mass ratio, *CVD* Cardiovascular disease, *DXA* Dual-energy X-ray absorptiometry, *CT* Computed tomography, *PI* Protease inhibitor, *NNRTI* Non-nucleoside reverse transcriptase inhibitor, *NRTI* Nucleoside reverse transcriptase inhibitor, *VAT* Visceral adipose tissue, *SAT* Subcutaneous adipose tissue, *HOMA* The homeostasis model assessment of insulin resistance, *HDL* High density cholesterol, *LDL* Low density cholesterol, *Apo A1* Apolipoprotein A1, *Apo B* Apolipoprotein B, *Lp(a)* Lipoprotein (a), *CPR* C protein reactive, *hsCRP* High sensitivity C protein reactive, *NT-ProBNP* N-terminal prohormone of brain natriuretic peptide, *IMT* Intima-media thickness)

Patients with lipodystrophy had a lower value of hip circumference and a higher waist/hip circumference ratio, compared to the patients without lipodystrophy. (*p* = 0.006 and 0.003, respectively).

No differences were observed between patients with, and without lipodystrophy regarding viral suppression rate, HIV risk factor, CDC classification and ART regimen, smoking history, family history of cardiovascular disease and medication history (statins, fibrates, oral anti-diabetics, insulin and anti-hypertensive drugs), systolic blood pressure, and CD4+ cell count.

Regarding the assessment of body composition by DXA, patients with lipodystrophy presented lower values of total and leg fat mass (*p* = 0.017 and <0.001, respectively), both in % and Kg (Table 1). No differences were observed in trunk and arm fat mass (in % and Kg) between the two groups.

Patients with lipodystrophy had a higher visceral adipose tissue (VAT) value and VAT/SAT ratio (*p* = 0.011 and 0.002, respectively), lower subcutaneous adipose tissue (SAT) (*p* = 0.011), and no significant difference in total fat at abdominal level.

No significant differences were found in the levels of glucose, insulin, lipid profile (total cholesterol, LDL, HDL, non-HDL cholesterol and triglycerides), uric acid, lactates, HOMA, HbA1c, Apo A1, Apo B, ratio Apo B/Apo A1, Lp(a), homocysteine, CRP, hsCRP, NT-proBNP, fibrinogen, and microalbumin urinary excretion between patients with or without lipodystrophy.

Furthermore, no significant differences regarding the prevalence of metabolic syndrome were observed between the two groups (Table 1).

#### Carotid IMT measurements

Carotid IMT was higher in patients with lipodystrophy, compared with those without lipodystrophy [mean (SD) 0.82 (0.26) vs. 0.73 (0.20); *p* = 0.050] (Table 1).

The cIMT was positively correlated with lipodystrophy evaluated by FMR, age, neck circumference, waist/hip ratio, systolic blood pressure, glucose at 0 and 120 min on OGTT, HbA1c, non-HDL cholesterol, visceral obesity defined by total body fat mass by quantitative CT, VAT and VAT/SAT ratio, trunk fat mass evaluated by DXA, uric acid, CRP, hsCRP, and homocysteine, as is shown in Table 2. No significant correlations were found between cIMT and duration of HIV infection, length of cART, thigh circumference, total, leg and arm fat mass evaluated by DXA, CD4 cell count, leukocyte, insulin at 0 and 120 min on OGTT, HOMA, triglycerides, and lactates.

**Table 2:** Associations between cIMT and body composition, as well as metabolic parameters

Correlations between cIMT and:	R	*P*
Age	0.709	<0.001
Waist/Hip Ratio	0.387	<0.001
Neck circumference	0.202	0.007
Systolic Blood Pressure	0.419	<0.001
Glucose 0 min	0.284	<0.001
Glucose 2 h OGTT	0.220	0.006
A1c	0.285	<0.001
Non-HDL cholesterol	0.254	<0.001
CRP	0.196	0.005
hsCRP	0.334	0.003
Homocysteine	0.150	0.032
Uric Acid	0.146	0.034
Trunk Fat Mass by DXA (g)	0.168	0.0018
Total fat on Abdominal CT scan	0.218	0.002
VAT on abdominal CT scan	0.418	<0.001
VAT/SAT on abdominal CT scan	0.290	<0.001
FMR	0.250	<0.001
Duration of HIV infection	−0.019	0.779
Leg Fat Mass by DXA (g)	−0.037	0.604
Thigh Circumference	−0.095	0.169
CD4 cell count	−0.027	0.701
Leukocytes	−0.049	0.478
SAT on abdominal CT scan	−0.009	0.901
Insulin 2 h OGTT	−0.089	0.271
HOMA	0.094	0.185
Insulin 0 min	0.026	0.711
Lactates	0.081	0.253
Triglycerides	0.109	0.113
Total Fat Mass by DXA (g)	0.084	0.236
Arm Fat Mass by DXA (g)	0.029	0.686
cART	0.024	0.727

(*FMR* Fat mass ratio, *OGTT* Oral glucose tolerance test, *CT* Computed tomography, *VAT* Visceral adipose tissue, *SAT* Subcutaneous adipose tissue, *HDL* High density cholesterol, *CPR* C protein reactive; hsCRP High sensitivity C protein reactive, *HOMA* The homeostasis model assessment of insulin resistance, *DXA* dual-energy X-ray absorptiometry, *cART* Combined antiretroviral therapy, *IMT* Intima-media thickness)

The progression of the study sample’s characteristics throughout the period of follow-up (12 months) is illustrated in Table 3.

**Table 3:** Progression of sample characteristics during follow-up

	Total Population			With Lipodystrophy			Without Lipodystrophy		
	Baseline	1 year after	*p*	Baseline	1 year after	*P*	Baseline	1 year after	*p*
Weight [Kg, mean (sd)]	68.7 (11.9)	67.8 (11.8)	0.474	67.8 (9.6)	65.7 (12.0)	0.360	69.5 (13.5)	69.5 (13.5)	0.977
Height [m, mean (sd)]	1.65 (0.1)	1.65 (0.1)	–	1.64 (0.08)	1.64 (0.08)	–	1.66 (0.09)	1.66 (0.09)	–
BMI [(Kg/m^2^), mean (sd)]	25.2 (4.3)	25.0 (4.9)	0.547	25.2 (3.8)	24.5 (4.7)	0.359	25.2 (4.7)	25.4 (5.1)	0.849
Waist circumference [cm, mean (sd)]	91.4 (11.5)	90.9 (10.8)	0.654	91.6 (9.9)	89.7 (11.2)	0.402	91.4 (12.8)	92.0 (10.4)	0.804
Hip circumference [cm, mean (sd)]	94.0 (7.8)	93.2 (6.8)	0.496	91.9(6.4)	92.6 (7.0)	0.561	95.7(8.6)	93.6 (6.7)	0.120
Thigh circumference [cm, mean (sd)]	48.0 (5.2)	45.3 (4.2)	<0.001	47.2 (5.2)	45.0 (4.14)	0.023	48,7 (5.1)	45.5 (4.4)	<0.001
Arm circumference [cm, mean (sd)]	27.0 (2.9)	26.1 (2.8)	0.049	27.0 (2.7)	25.9 (2.9)	0.082	27.0 (3.1)	26.5 (2.8)	0.353
Neck circumference [cm, mean (sd)]	37.7 (3.5)	36.9 (3.5)	0.143	37.6 (3.3)	36.8 (3.9)	0.327	37.8 (3.6)	37.1 (3.2)	0.322
Waist/ hip circumference ratio [mean (sd)]	0.97 (0.08)	0.97 (0.08)	0.786	1.00 (0.07)	0.97 (0.09)	0.086	0.95 (0.09)	0.98 (0.08)	0.066
Fat mass [%, mean (sd)] DXA									
Total	20.5 (11.4)	21.6 (10.4)	0.554	17.8 (8.0)	19.9 (10.5)	0.264	22.6 (13.1)	22.9 (10.1)	0.941 0.418
Trunk	23.5 (11.6)	24.8 (10.8)	0.561	22.7 (8.6)	22.7 (11.2)	0.980	24.1 (13.5)	26.5 (10.2)	
Leg	16.7 (13.8)	16.2 (11.0)	0.898	9.7 (6.7)	15.0 (10.4)	0.007	22.3 (15.4)	17.2 (11.5)	0.088
Arm	21.3 (15.2)	23.8 (15.0)	0.267	18.7(13.3)	22.4 (15.8)	0.232	23.4 (16.3)	24.9 (14.4)	0.631
Fat mass [Kg, mean (sd)] DXA									
Total	14.5 (9.5)	14.7 (8.3)	0.867	12.1 (6.7)	13.1 (7.7)	0.406	16.4 (11.0)	16.0 (8.6)	0.736
Trunk	8.7 (5.4)	9.0 (5.0)	0.897	8.4 (4.3)	8.0 (4.9)	0.766	9.1 (6.2)	9.8 (5.1)	0.641
Leg	3.5 (3.0)	3.3 (2.5)	0.810	1.8 (1.3)	2.8 (2.0)	0.008	4.7 (3.4)	3.6 (2.8)	0.047
Arm	1.7 (1.5)	1.8 (1.3)	0.293	1.4 (1.2)	1.7 (1.3)	0.238	1.9 (1.7)	1.9 (1.3)	0.744
Body Fat Mass by Quantitative CT									
Total fat [cm^2^, mean (sd)]	266.8 (158.4)	274.5 (146.0)	0.625	259.6 (112.9)	249.6 (160.5)	0.708	272.6 (188.1)	295.3 (130.4)	0.361
VAT [cm^2^, mean (sd)]	133.8 (83.2)	136.0 (85.1)	0.670	154.8 (59.7)	126.2 (97.2)	0.049	116.8 (95.4)	144.2 (73.3)	0.024
SAT [cm^2^, mean (sd)]	133.0 (110.4)	139.3 (95.7)	0.314	104.9 (85.2)	125.2 (95.8)	0.180	155.8 (123.2)	151.1 (94.8)	0.848
VAT/SAT ratio [cm^2^, mean (sd)]	1.8 (1.8)	1.6 (1.8)	0.298	2.4 (1.7)	1.6 (1.6)	0.019	1.3 (1.8)	1.6 (1.9)	0.185
Systolic blood pressure [mmHg, mean (sd)]	122.9 (18.0)	117.2 (18.1)	0.005	124.0 (16.1)	119.3 (20.9)	0.072	122.0 (19.4)	115.6 (15.5)	0.027
Diastolic blood pressure [mmHg, mean (sd)]	78.8 (11.6)	71.8 (8.9)	<0.001	79.2 (11.1)	72.2 (8.6)	0.001	78.5 (12.1)	71.6 (9.2)	<0.001
Glucose 0 min [mg/dL, mean (sd)]	112.0 (49.6)	101.2 (25.6)	0.481	119.1 (52.1)	104.6 (28.7)	0.307	106.4 (47.1)	99.4 (22.8)	0.992
Glucose 2 h [mg/dL, mean (sd)]	129.5 (47.4)	137.3 (47.6)	0.445	138.5 (47.7)	137.7 (47.5)	0.819	122.3 (46.3)	136.9 (48.2)	0.451
Insulin 0 min [μU/mL, mean (sd)]	10.7 (10.4)	13.3 (14.8)	0.166	10.9 (6.5)	12.1 (9.9)	0.419	10.5 (12.8)	14.1 (17.7)	0.227
Insulin 2 h [μU/mL, mean (sd)]	76.5 (131.5)	71.8 (60)	0.886	105.9 (186.8)	69.9 (60.4)	0.220	53.1 (49.2)	73.3 (59.9)	0.307
HOMA [mean (sd)]	3.1 (3.2)	3.5 (4.8)	0.552	3.2 (2.5)	3.2 (2.9)	0.857	2.9 (3.6)	3.8 (5.8)	0.369
A1c [% mean (sd)]	5.8 (1.2)	5.5 (0.8)	0.095	5.9 (1.1)	5.5 (0.9)	0.096	5.6 (1.2)	5.4 (0.7)	0.556
Total cholesterol [mg/dL, mean (sd)]	219.5 (56.8)	204.7 (41.4)	0.012	226 (57.6)	203.8 (41.9)	0.007	213.6 (56.0)	205.4 (41.2)	0.396
LDL-cholesterol [mg/dL, mean (sd)]	118.0 (49.2)	135.7 (31.3)	0.001	123.7 (49.2)	133.9 (28.4)	0.153	113.5 (49.2)	137.2 (33.6)	0.001
HDL-cholesterol [mg/dL, mean (sd)]	43.8 (11.7)	48.6 (13.3)	0.012	43.6 (11.3)	50.6 (14.6)	0.018	43.9 (12.1)	46.9 (12.0)	0.280
Non-HDL cholesterol [mg/dL, mean (sd)]	175.7 (52.3)	156.2 (37.6)	<0.001	183.2 (52.2)	153.2 (37.6)	<0.001	169.8 (52.0)	158.5 (37.6)	0.192
Triglycerides [mg/dL, mean (sd)]	290.8 (189.0)	180.5 (110.5)	<0.001	298.9 (186.9)	165.6 (107.2)	<0.001	284.4 (191.7)	192.3 (112.5)	0.001
Apo A1 [mg/dL, mean (sd)]	111 (21)	126.4 (24.0)	<0.001	111.6 (16.6)	130.0 (25.2)	0.001	110.4 (23.9	123.7 (22.8)	0.002
Apo B [mg/dL, mean (sd)]	104.7 (25.3)	101.4 (21.7)	<0.001	104.8 (26.7)	100.0 (21.6)	0.126	104.7 (24.4)	102.4 (22.0)	0.706
Ratio apo B/apo A1 [mean (sd)]	0.97 (0.3)	0.83 (0.3)	<0.001	0.95 (0.3)	0.79 (0.2)	0.001	0.98 (0.3)	0.87 (0.3)	0.02
Lp (a) [mg/dL, mean (sd)]	28.3 (32.0)	36.3 (43.3)	0.289	27.5 (33.5	44.4 (47.8)	0.102	28.9 (30.9)	30.1 (38.7)	0.906
Homocysteine [μmol/L, mean (sd)]	9.5 (3.8)	11.1 (4.8)	0.005	9.7 (3.5)	12.1 (5.2)	0.008	9.4 (4.1)	10.3 (4.3)	0.179
CRP [mg/L, mean (sd)]	5.6 (12.8)	5.1 (11.3)	0.147	4.3 (4.7)	6.1 (14.4)	0.857	6.6 (16.6)	4.3 (8.0)	0.038
hsCRP [mg/dL, mean (sd)]	0.6 (1.2)	0.5 (1.2)	0.030	0.34 (0.31)	0.65 (1.6)	0.225	0.83 (1.5)	0.43 (0.8)	0.066
Lactates [mmol/L, mean (sd)]	1.2 (0.5)	1.4 (0.6)	0.002	1.3 (0.6)	1.4 (0.6)	0.307	1.1 (0.48)	1.4 (0.5)	0.001
NT-ProBNP [pg/mL, mean (sd)]	36.8 (59.0)	39.0 (63.0)	0.241	31.6 (31.9)	35.9 (33.2)	0.177	41.2 (74.8)	41.3 (79.0)	0.769
Fibrinogen [mg/dL, mean (sd)]	347.3 (104.4)	332.6 (79,0)	0.611	342.9 (105.9)	333.8 (80.1)	0.841	350.8 (103.8)	331.6 (78.8)	0.370
Microalbumin [mg/L, mean (sd)]	58.2 (151.4)	36.0 (159.8)	0.527	40.6 (91.3)	20.5 (35.6)	0.551	76.7 (196.6)	47.7 (209.3)	0.852
Uric acid [mg/L, mean (sd)]	47.7 (14.0)	44.1 (14.6)	0.022	49.8 (12.8)	43.7 (13.0)	0.005	45.9 (14.6)	474.3 (15.8)	0.470
Carotid IMT [mm, mean (sd)]	0.77 (0.23)	0.87 (0.32)	0.001	0.82 (0.26)	0.92 (0.33)	0.037	0.73 (0.20)	0.84 (0.30)	0.012
Metabolic Syndrome (%)	58 (51.3)	48 (43.2)	0.203	27 (54.0)	19 (38.8)	0.134	31 (49.2)	29 (46.8)	0.851

(L- Lipodystrophy FMR defined; *BMI* Body mass index, *FMR* Fat mass ratio, *DXA* Dual-energy X-ray absorptiometry, *CT* Computed tomography, *VAT* Visceral adipose tissue, *SAT* Subcutaneous adipose tissue, *HOMA* The homeostasis model assessment of insulin resistance, *HDL* High density cholesterol, *LDL* Low density cholesterol, *Apo A1* Apolipoprotein A1, *Apo B* Apolipoprotein B, *Lp(a)* Lipoprotein (a), *CPR* C protein reactive, *hsCRP* High sensitivity C protein reactive, *NT-ProBNP* N-terminal prohormone of brain natriuretic peptide *IMT* Intima-media thickness)

### One year follow-up

#### Total population

With regards to the progression of body composition and metabolic parameters during one year in the total of the sample, the values of thigh and arm circumference are lower than those obtained at baseline (*p* < 0.001 and 0.049, respectively). The systolic and diastolic blood pressure also decreased significantly in the follow-up period (*p* = 0.005 and <0.001), as well as total cholesterol, non-HDL cholesterol, and triglycerides levels (*p* = 0.012 and <0.001, for both non-HDL cholesterol and triglycerides). The values of HDL, LDL and Apo A1 increased since the baseline (*p* = 0.012, 0.001 and <0.001, respectively), and the values of Apo B and the ratio apo B/ apo A1 decreased (*p* < 0.001 for both variables).

Lastly, values of homocysteine and lactates were higher (*p* = 0.005 and 0.002), when compared with the baseline, and the values of hsCRP and uric acid were lower (*p* = 0.030 and 0.022).

#### Patients with Lipodystrophy

Patients with lipodystrophy presented lower thigh circumference when compared with the baseline (*p* = 0.023), but higher values of leg fat mass, both in Kg and % (*p* = 0.008 and 0.007, respectively). The analysis according to quantitative CT showed a decreased value of VAT and VAT/SAT ratio (*p* = 0.049 and 0.019). Diastolic blood pressure, total cholesterol, non-HDL cholesterol and triglycerides levels were lower compared with baseline values (*p* = 0.001, 0.007, and <0.001 for both non HDL-cholesterol and triglycerides levels). Furthermore, HDL and Apo A1 levels increased (*p* = 0.018 and 0.001), and the Apo B/Apo A1 ratio decreased (*p* = 0.001). The values of homocysteine were higher, while uric acid levels were lower than the ones obtained at the baseline (*p* = 0.008 and 0.005, respectively).

#### Patients without Lipodystrophy

Patients without lipodystrophy presented lower thigh circumference and leg fat mass (Kg) compared with the baseline (*p* < 0.001 and 0.047). The value of VAT increased in this group during the follow-up period (*p* = 0.024). In addition to the decline of systolic and diastolic blood pressure (*p* = 0.027 and <0.001, respectively), CRP and hsCRP levels also decreased (*p* = 0.038 and 0.066). The values of LDL, Apo A1 and lactates increased (*p* = 0.001, 0.002 and 0.001), while triglycerides levels and Apo B/Apo A1 ratio decreased (*p* = 0.001 and 0.02, respectively).

#### Carotid IMT measurements

During one year of follow-up there was an increase in carotid IMT in the total of HIV-infected patients and in those with and without lipodystrophy, when compared with the baseline (*p* = 0.001, 0.037 and 0.012, respectively), and this remained higher in patients presenting lipodystrophy when compared to those without lipodystrophy, although without significant differences between them (*p* = 0.178).

Table 2 shows the variables that were positively correlated with carotid IMT at the baseline. The one year evolution of these variables during follow-up in the total population can be observed in Table 4. During the follow-up period, the cIMT increased in the totality of the sample [0.77(0.23) and 1 year after 0.87 (0.32) mm; *p* = 0.001], despite the significant decrease in the values of systolic blood pressure [122.9 (18.0) and 1 year after 117.2 (18.1) mmHg; *p* = 0.005], diastolic blood pressure [78.8 (11.6) and 1 year after 71.8 (8.9) mmHg; *p* < 0.001], total cholesterol [219.5 (56.8) and 1 year after 204.7 (41.4) mg/dL; *p* = 0.012], non-HDL cholesterol [175.7 (52.3) and 1 year after 156.2 (37.6) mg/dL; p < 0.001], triglycerides [290.8 (189.0) and 1 year after 180.5 (110.5) mg/dL; p < 0.001], apo B [104.7 (25.3) and 1 year after 101.4 (21.7) mg/dL; p < 0.001], hsCRP [0.6 (1.2) and 1 year after 0.5 (1.2) mg/dL; *p* = 0.030] and uric acid [47.7 (14.0) and 1 year after 44.1 (14.6) mg/L; *p* = 0.022]. The only variable positively correlated with cIMT that significantly increased in the total population was homocysteine.

**Table 4:** Evolution of the variables positively correlated with cIMT during follow-up in the total sample

	Baseline	One Year follow-up	*P*
Waist/Hip Ratio [mean (sd)]	0.97 (0.08)	0.97 (0.08)	0.786
Neck circumference [cm, mean (sd)]	37.7 (3.5)	36.9 (3.5)	0.143
Systolic Blood Pressure [mmHg, mean (sd)]	122.9 (18.0)	117.2 (18.1)	0.005
Glucose 0 min [mg/dL, mean (sd)]	112.0 (49.6)	101.7 (25.6)	0.481
Glucose 2 h OGTT [mg/dL, mean (sd)]	129.5 (47.4)	137.3 (47.6)	0.445
A1c [% mean (sd)]	5.8 (1.2)	5.5 (0.8)	0.095
Non-HDL cholesterol [mg/dL, mean (sd)]	175.7 (52.3)	156.2 (37.6)	<0.001
CRP [mg/L, mean (sd)]	5.6 (12.8)	5.1 (11.3)	0.147
hsCRP [mg/dL, mean (sd)]	0.6 (1.2)	0.5 (1.2)	0.030
Homocysteine [μmol/L, mean (sd)]	9.5 (3.8)	11.1 (4.8)	0.005
Uric Acid [mg/L, mean (sd)]	47.7 (14.0)	44.1 (14.6)	0.022
Trunk Fat Mass by DXA (kg) [Kg, mean (sd)]	8.7 (5.4)	9.0 (5.0)	0.897
Total fat on Abdominal CT scan [cm^2^, mean (sd)]	266.8 (158.4)	274.5 (146.0)	0.625
VAT on abdominal CT scan [cm^2^, mean (sd)]	133.8 (83.2)	136.0 (85.1)	0.670
VAT/SAT on abdominal CT scan [cm^2^, mean (sd)]	1.8 (1.8)	1.6 (1.8)	0.298

*OGTT* Oral glucose tolerance test, *CT* Computed tomography, *VAT* Visceral adipose tissue, *SAT* Subcutaneous adipose tissue, *HDL* High density cholesterol, *CPR* C protein reactive, *hsCRP* High sensitivity C protein reactive, *DXA* Dual-energy X-ray absorptiometry, *IMT* Intima-media thickness)

Using generalised linear models for repeated measures, means of cIMT between the baseline and the 1 year follow-up were calculated and adjusted for age, glucose, triglycerides levels, systolic blood pressure and waist to hip ratio (Table 5). In the total population, significantly higher mean values of cIMT were observed after the one year follow-up, after adjustment for age [0.770 (0.738–0.802) basal vs 0.874 (0.816–0.932 1 year after, <0.001], for age and glucose [0.770 (0.737–0.803) basal vs 0.874 (0.816–0.932) 1 year after; *p* < 0.001], for age, glucose and triglycerides [0.770 (0.737–0.803) basal vs 0.874 (0.815–0.933) 1 year after; *p* = 0.001] and for age, glucose, triglycerides, and systolic blood pressure [0.770 (0.738–0.802) basal vs 0.874 (0.815–0.932) 1 year after; *p* = 0.050]. However, after further adjustment for age, glucose, triglycerides, systolic pressure, and waist/hip ratio, this difference did not remain statistically significant[0.770 (0.737–0.803) basal vs 0.874 (0.815–0.933) 1 year after; *p* = 0.514].

**Table 5:** Crude and adjusted means of cIMT at baseline and at 1 year follow up

Total Sample	cIMT baseline	cIMT 1 year follow up	*P*
Crude model	0.770 (0.728–0.812)	0.874 (0.815–0.932)	<0.001
Model adjusted for age	0.770 (0.738–0.802)	0.874 (0.816–0.932)	<0.001
Model adjusted for age and glucose	0.770 (0.737–0.803)	0.874 (0.816–0.932)	<0.001
Model adjusted for age, glucose and Triglycerides	0.770 (0.737–0.803)	0.874 (0.815–0.933)	0.001
Model adjusted for age, glucose, triglycerides and Systolic Pressure	0.770 (0.738–0.802)	0.874 (0.815–0.932)	0.050
Model adjusted for age, glucose, triglycerides, Systolic Pressure and WHR	0.770 (0.737–0.803)	0.874 (0.815–0.933)	0.514
Patients with Lipodystrophy			
Crude model	0.817 (0.744–0.889)	0.919 (0.825–1.01)	0.030
Model adjusted for age	0.817 (0.757–0.876)	0.919 (0.826–1.01)	0.027
Model adjusted for age and glucose	0.817 (0.756–0.877)	0.919 (0.825–1.01)	0.018
Model adjusted for age, glucose and triglycerides	0.817 (0.756–0.877)	0.919 (0.825–1.01)	0.053
Model adjusted for age, glucose, triglycerides and Systolic Pressure	0.817 (0.756–0.878)	0.919 (0.825–1.01)	0.401
Model adjusted for age, glucose, triglycerides, Systolic Pressure and WHR	0.817 (0.755–0.878)	0.919 (0.823–1.01)	0.644
Patients Without Lipodystrophy			
Crude model	0.733 (0.684–0.784)	0.838 (0.763–0.914)	0.020
Model adjusted for age	0.733 (0.698–0.767)	0.838 (0.763–0.914)	0.003
Model adjusted for age and glucose	0.733 (0.698–0.768)	0.838 (0.763–0.914)	0.015
Model adjusted for age, glucose and triglycerides	0.733 (0.698–0.768)	0.838 (0.762–0.915)	0.017
Model adjusted for age, glucose, triglycerides and Systolic Pressure	0.733 (0.698–0.768)	0.838 (0.762–0.915)	0.130
Model adjusted for age, glucose, triglycerides, Systolic Pressure and WHR	0.733 (0.698–0.768)	0.838 (0.761–0.915)	0.676

(L- Lipodystrophy FMR defined; *FMR* Fat mass ratio, *SBP* Systolic blood pressure, *WHR* Waist/hip ratio, *IMT* Intima-media thickness)

The same analysis was performed stratifying for the presence of lipodystrophy, and higher mean adjusted values of cIMT [0.817 (0.744–0.889) vs 0.919 (0.825–1.01) 1 year after; *p* = 0.030], adjusted for age [0.817 (0.757–0.876) vs 0.919 (0.826–1.01); *p* = 0.027)], age and glucose [0.817 (0.756–0.877) vs 0.919 (0.825–1.01); *p* = 0.018], and age, glucose and triglycerides [0.817 (0.756–0.877) vs 0.919 (0.825–1.01); *p* = 0.053) were observed at one year of follow-up, but this difference lost its statistical significance after further adjustment for systolic blood pressure [0.817 (0.756–0.878) vs 0.91 (0.825–1.01); *p* = 0.401), and waist/hip ratio [0.817 (0.755–0.878) vs 0.919 (0.823–1.01); *p* = 0.644]. The same results were observed for patients without lipodystrophy (Table 5).

## Discussion

Atherosclerosis is a progressive disease, and its contribution to cardiovascular risk increase is well-known [21]. This conventional CVD risk factor, as well as body fat redistribution, is associated with the progression of cIMT [22]. The intervention in cardiometabolic risk factors through the introduction of changes in terms of diet, exercise, smoking cessation, and drug therapy (antidiabetic, antihypertensive and anti-dyslipidemic) may regress or delay the progression of atherosclerosis [23]. We evaluated the progression of cIMT at the end of the one year follow-up in a cohort of HIV-infected patients under cART, treated for the conventional risk factors. Indeed, after a year of follow-up, patients improved some lipid profile components and blood pressure levels, and showed decreased VAT values. Despite these improvements, mean values of cIMT increased during this period of time. In fact, after one year, HIV-infected patients (with and without lipodystrophy) had a significant progression of cIMT, even after adjustment for some conventional cardiovascular risk factors (age, glucose and triglycerides). However, during the follow-up, we observed an increase in LDL in the total of the population and also in those without lipodystrophy, which could impact on the progression of cIMT.

Furthermore, the presence of lipodystrophy defined by FMR was not associated with the progression of cIMT. Our data suggest that the increase of cIMT is associated with body fat abnormalities, particularly visceral body fat. It has been established that cART can cause a wide spectrum of metabolic disturbances and abnormalities in body fat distribution, and its contribution to premature and accelerated atherosclerosis in HIV infected patients is well-known [10, 24, 25]. Since our main goal was to determine whether the progression of cIMT was associated with lipodystrophy, our sample only included HIV infected patients under cART. The overall progression of cIMT in both groups (with and without lipodystrophy) may reflect the fact that all the patients were subjected to cART and all were under a low-grade chronic state of inflammation associated with the HIV disease per se.

As pointed out above, the mean values of cIMT increased significantly in both groups of the population, despite the fact that, at the baseline, lipodystrophic patients had a higher waist/hip circumference ratio, VAT and VAT/SAT ratio. At follow-up, on the other hand, this group had lower WHR values, but showed equivalent results for cardiometabolic markers, when compared with patients without lipodystrophy. During the one year of follow-up, the values for VAT, VAT/SAT ratio, and WHR did not change significantly in the total of the population, and neither in those with and without lipodystrophy. Analyses from large observational cohorts have demonstrated that cIMT increase ranging from 0.03 to 0.2 mm are associated with 33% to 300% higher risk of coronary heart disease and stroke [26–28]. In our study, the progression of cIMT was over 0.1 mm in both groups, thus reflecting an increased cardiovascular risk in HIV infected patients under cART.

The degree to which cART therapy contributes to the increased cardiovascular risk in HIV patients is not completely clear yet. Even though there is a large body of evidence in the literature pointing to a moderate association between HIV infection and increased cIMT, in both cross-sectional and prospective studies, the results are still controversial [29–31]. A prospective study that evaluated the progression of cIMT in HIV-infected patients during 6 years found that HIV-infected patients accumulate CVD risk over time, beyond that experienced in the general population [32]. A recent large prospective cohort study that evaluated the progression of subclinical atherosclerosis in HIV-infected patients, over seven years, concluded that HIV infection is associated with increased cardiovascular risk and that this elevated risk persisted among cART-treated individuals with persistent HIV viral suppression [33]. Hanna et al. showed that HIV infection is associated with greater increases in focal plaque formation, even when the patients are subjected to antiretroviral therapy, but that HIV infection itself was not associated with increased cIMT over time, and no associations were found between persistent virologic suppression and cIMT progression, compared with uninfected individuals [33]. These findings contradict several other studies that reported an association between HIV infection and cIMT progression over time [5, 32, 34–37]. Although van Vonderen et al. reported an independent association between HIV infection and increased cIMT, cART use was only associated with increased stiffness of the femoral artery, not cIMT [5]. The same study also did not find an independent association between lipodystrophy and cIMT [5], which is consistent with our study. Volpe et al. found that changes in cIMT over time were associated not only with tradition risk factors, but also with HIV-related factors and that these associations vary with the type of surrogate marker evaluated [30]. In fact, some studies have suggested that traditional risk factors overshadow the role of HIV infection and cART use, and that they are the major influences of cIMT progression [6, 37–39]. Visceral obesity is a well-known independent risk factor for CVD in terms of the population as a whole, [40] and independent associations between carotid intima-media thickness and abdominal adiposity were already demonstrated [41]. Although the physiologic mechanisms by which obesity is linked to an early carotid atherosclerosis are not clearly described, some theories regarding the importance of metabolic factors, such as changes in plasma adiponectin levels and insulin resistance, have been proposed [6]. In our study, we found a higher prevalence of visceral adiposity among patients with lipodystrophy. We expected that the visceral adiposity present in patients with lipodystrophy would contribute to the progression of cIMT, which was not the case. We can speculate that the high frequency of visceral adiposity in patients without lipodystrophy defined by FMR may contribute to the absence of differences in the progression of cIMT between patients with and without lipodystrophy. In contrast with the results obtained at baseline, the mean values of cIMT remained significant after adjustment for age. In fact, the progression of cIMT remained significant after adjustment for age, glucose, triglycerides and systolic blood pressure, but statistical significance was lost when the association was further adjusted for the waist/hip ratio. This indicates that although cIMT is known to be affected by age, in our study this was not the case, as, in fact, cIMT progression was mostly affected by the presence of visceral adiposity.

Additional research, with a large sample-size, is needed to better characterize the underlying proatherogenic mechanisms of HIV infection and cART, as well as to evaluate the impact of body composition, namely lipodystrophy, on long-term cardiovascular risk in HIV-infected patients.

### Study limitations

The limitations of this study are mainly related to the small size of the sample. Therefore, this study might have been underpowered for the detection of small differences in cIMT progression between the two groups. Due to the fact that the design of this study did not include an HIV-uninfected control group, it remains unclear whether the observed increased cardiovascular risk can be fully explained by the effects of antiretroviral drugs and their metabolic side effects, body composition, or whether chronic HIV infection itself may play a role. Another limitation of our study is the absence of data regarding the exposure time to each drug, since we only have information on the current class of antiretroviral, thus the impact of the duration of cART regimen could not be assessed in this study.

### Highlights of this study

This study emphasizes the role of body composition, namely VAT, in addition to other traditional risk factors in the progression of cIMT. Moreover, it was performed in a unit that is highly experienced in the assessment of metabolic and body fat abnormalities in HIV-infected patients. All clinical evaluations were carried out by the same practitioner, and an objective definition of lipodystrophy was used (fat mass ratio by DXA).

## Conclusions

Carotid IMT progressed significantly in HIV-infected patients under cART, both in those with and without lipodystrophy. Although patients with lipodystrophy defined by FMR had a higher cIMT when compared with those without lipodystrophy, there was no association between the progression of cIMT and the presence of lipodystrophy defined by FMR. Visceral adipose tissue had an impact on the increment of cIMT during the follow-up period (both in patients with and without lipodystrophy defined by FMR), which suggests an independent association between VAT and the increasing of subclinical carotid atherosclerosis.

## Abbreviations

cART: Combination antiretroviral therapy
cIMT: Carotid intima-media thickness
CVD: Cardiovascular disease
FMR: Fat Mass Ratio
L-FMR: Lipodystrophy defined by FMR
OGTT: Oral glucose tolerance test
SAT: Subcutaneous adipose tissue
SPB: Systolic blood pressure
VAT: Visceral adipose tissue
WHR: Waist/hip ratio
